# Liquid-Exfoliated Antimony Nanosheets Hybridized with Reduced Graphene Oxide for Photoelectrochemical Photodetectors

**DOI:** 10.3390/nano15171355

**Published:** 2025-09-03

**Authors:** Gengcheng Liao, Sichao Yu, Jiebo Zeng, Zongyu Huang, Xiang Qi, Jianxin Zhong, Long Ren

**Affiliations:** 1 Institute for Quantum Science and Technology, Shanghai University, Shanghai 200444, China; 2 Hunan Key Laboratory of Micro-Nano Energy Materials and Devices, Laboratory for Quantum Engineering and Micro-Nano Energy Technology, School of Physics and Optoelectronic, Xiangtan University, Xiangtan 411105, China; 3 Hunan Key Laboratory of Two Dimensional Materials, Hunan University, Changsha 410082, China

**Keywords:** Sb nanosheets, photoelectrochemical, photodetectors

## Abstract

In this paper, we design a self-powered photoelectrochemical (PEC)-type photodetector based on a hybridization of two-dimensional (2D) few-layer antimony (Sb) nanosheets (NSs) and reduced graphene oxide (rGO). The few-layer Sb NSs obtained by liquid-phase exfoliation can be anchored on the surface of rGO through hydrothermal treatment. Specifically, during photoexcitation, the electron–hole pairs photogenerated on the surface of Sb NSs can be well stimulated and transferred by rGO, reducing the photogenerated carriers recombine on Sb NSs. The excellent electrochemical performance is confirmed by PEC tests. The photobehavior performance of the Sb NSs-rGO composite is significantly improved; its photocurrent density reaches 48.830 nA/cm^2^ at zero potential, approximately twice that of pure Sb NSs. The hybrid exhibits a faster photoresponse speed, with the response time and recovery time being 0.140 s and 0.163 s, respectively. This enhancement arises from the conductive role of rGO as a conductive channel, and as a result, the efficient separation of photoinduced electron–hole pairs is facilitated. This study is a further exploration of hybrid engineering of 2D materials in photochemical photodetectors and demonstrates significant progress in this field.

## 1. Introduction

Over the past few years, the emergence of two-dimensional (2D) materials has significantly accelerated global research [1,2,3]. These 2D materials have garnered significant attention in optoelectronic device applications because of their exceptionally thin-layered configurations and outstanding characteristics [4]. As a novel 2D layered material with few atomic layers, antimony (Sb) NSs exhibits superior photoresponse and stability [5,6], which have demonstrated a profound overall electrochemical performance in water splitting [7], energy conversion [6], and N_2_ fixation [8]. In addition, in the field of optoelectronics, Sb NSs exhibit significant advantages of a high photoelectric response performance and fast response speed. This is due to the excellent wide spectral absorption characteristics of Sb NSs, which can cover the range from visible light to infrared light [8,9]. At the same time, its ultra-thin structure significantly shortens the transmission path of charge carriers, thereby greatly improving the response speed [10,11]. However, its photo-excited photo-generated charge carriers will rapidly recombine, which limits its further development in the field of photochemical photodetectors [12].

With the escalating demands of photoelectric devices, it is obvious that a single material cannot meet the needs of high-performance photoelectronic devices. Designing heterojunction systems has been proven to be an efficient way to boost the efficiency of separated photogenerated carriers [13,14,15]. Among all, 2D graphene has demonstrated significant potential for use in heterojunction engineering because of its high carrier mobility, great conductivity, and tremendous surface area. It has been reported that the charge transfer of 2D materials is greatly increased when 2D materials are combined with graphene due to the high electron mobility of 2D materials and the excellent electron conductive property of graphene [16]. Furthermore, many researches have also proved that graphene combined with other 2D layered materials is also a very effective way to achieve the special electronic and optoelectronic function of 2D materials [17,18].

Photochemical (PEC)-type photodetectors are a type of Schottky junction photovoltaic device that efficiently converts optical signals into electrical signals. Its superior photoelectric performance, miniaturized design, and self-powered characteristics enable its extensive application in optical communication imaging systems and a variety of photodetectors [19,20]. In this work, we successfully fabricated a Sb NSs-rGO hybrid via a facile liquid exfoliation and hydrothermal method designed as a PEC-type photodetector. Through liquid-phase exfoliation, few-layer Sb NSs are effectively separated from their bulk counterparts, thereby preserving their intrinsic lattice structure. This Sb NSs exhibits a smooth surface morphology, high crystallinity, and elongated forms. PEC tests have revealed that the enhanced PEC performance of the Sb NSs-rGO hybrid can be primarily attributed to the superior charge conduction capability of rGO, which facilitates efficient electron transport. The great interface between Sb NSs and rGO promotes effective separation of photoinduced carriers, which is a step indispensable to the holistic advancement of PEC efficiency.

## 2. Experimental Section

### 2.1. Materials Preparation

A mass of Sb with a purity of 99.999% was acquired from Aladdin Co., Ltd. (Beijing, China). GO was purchased from Aladdin Co. NMP (99.5%) and acetone were purchased from Aladdin Co., Inc. It is assumed that all additional reagents were of analytical grade, unless stated otherwise, and thus were ready for use with no need for additional purification.

### 2.2. Synthesis of Materials

The Sb nanosheets (NSs) were fabricated by employing a liquid phase exfoliation technique. In particular, a considerable amount of Sb (approximately 10 g) was initially ground for half an hour using an agate mortar and pestle. Subsequently, the sample is subsequently immersed in a 100 mL beaker containing 100 mL NMP solution and subjected to an ultrasonic cleaning procedure. Throughout this whole process, the temperature was maintained below 15 °C using an ice-water bath to prevent oxidation of the exfoliated Sb NSs. Under these conditions, the as-prepared Sb NSs were successfully obtained.

The Sb NSs and reduced rGO were synthesized via a hydrothermal approach, and graphene oxide (GO) was prepared using an enhanced Hummers method [21]. The graphene oxide (GO) used in this study was synthesized from graphite powder using a modified Hummers method. This method mainly involves using potassium permanganate (KMnO_4_) as the oxidizing agent to oxidize graphite in a mixture of concentrated sulfuric acid (H_2_SO_4_) and phosphoric acid (H_3_PO_4_). The entire process was carried out under strictly controlled low-temperature conditions. The resulting oxidized graphene was then subjected to repeated centrifugation and washing until it reached a neutral pH value. Finally, by peeling the sample, graphene can be obtained.

The reduced GO was dispersed in the glycol solution and thoroughly stirred for 15 min. Then, Sb NSs and graphene oxide were added to the dispersed rGO glycol solution. Once thoroughly stirred, the mixture was transferred to a 50 mL Teflon-lined autoclave and placed in a Muffle oven at 120 °C for 12 h. Upon cooling the sample to ambient temperature, it was subjected to three consecutive washes with acetone and ethanol to ensure that any residual glycol solution was removed. Subsequently, the Sb-NSs-rGO hybrid samples were obtained through vacuum freeze-drying.

### 2.3. Characterization

Scanning electron microscopy images were obtained using VEGA3 SBH from TESCAN, Brno, Czech Republic. Raman spectra were recorded on an Alpha 300R (Wotton under-Edge, Germany, from Witec Focus Innovations Company), which was equipped with a 532 nm He–Ne laser. High-resolution transmission electron microscopy was performed on a JEOL 200F thermal-field emission microscope (JEOL, Tokyo, Japan) operated at 200 KV. The UV–Vis absorption spectrum was measured by UV–Vis Spectrophotometer (UV-2600i, Shimadzu, Kyoto, Japan). TGA was carried out by NETZSCH-STA 449 F5 (NETZSCH-Gerätebau GmbH, Selb, Germany) in an N_2_ atmosphere from 30 to 680 °C. XRD was carried out by XRD-D8 Discover (Bruker, Ettlingen, Germany). One-chamber electron spectroscopy for XPS was carried out at the electrochemistry workstation CHI660D (CH Instruments, Inc., Shanghai, China) with a 350-W xenon lamp (Beijing Newbit Technology Co., Ltd., Beijing, China). HSX-F300 (Beijing Newbit Technology Co., Ltd., Beijing, China) was used for all the PEC tests in this work.

### 2.4. Electrochemical Measurements

The electrochemical parameters and other response parameters of the prepared Sb NSs-rGO hybrid were measured during the tests using a standard electrochemical workstation (CHI660D, Shanghai Chenhua, Shanghai, China) at a scan rate of 10 mV s^−1^ under irradiation with a 350 W xenon arc lamp (CHF-XM 350, Beijing Newbit Technology Co., Ltd., Beijing, China). To replicate solar irradiation, a Xenon lamp was employed as the light source. In the experimental setup, a NSs-rGO hybrid coated on ITO was utilized as the working electrode. A Pt plate electrode was employed as the counter electrode, and an Ag/AgCl electrode was used as the reference electrode in a three-electrode system. A 0.5 M Na_2_SO_4_ solution was used as the electrolyte solution.

## 3. Results and Discussion

**Figure 1:**
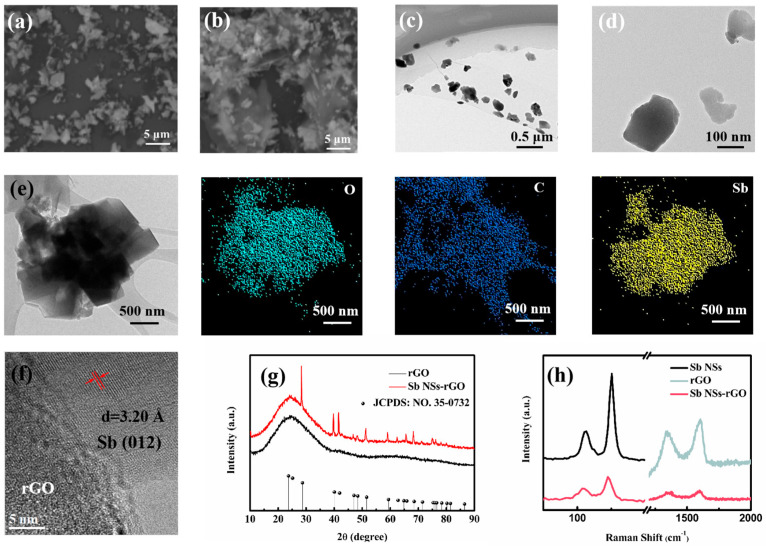
(**a**) SEM images of exfoliated Sb NSs and (**b**) as-prepared Sb NSs-rGO hybrid. (**c**) Low- and (**d**) high-resolution TEM images of as-prepared Sb NSs and (**e**) the as-prepared Sb NSs-rGO hybrid and its EDS mapping of O, C, and Sb. (**f**) Corresponding HRTEM images of Sb NSs-rGO hybrid. (**g**) XRD pattern of rGO and Sb NSs-rGO hybrid. (**h**) Raman spectra of Sb NSs, GO, and Sb NSs-rGO hybrid.

The morphological characteristics of the Sb nanosheets (NSs) revealed by scanning electron microscopy (SEM) are depicted in Figure 1a. Notably, the smooth surface and elongated layered structure are distinguishing features of the newly synthesized liquid-exfoliated Sb NSs. Figure 1b illustrates the morphological structure of the Sb NSs-rGO hybrid, where it is evident that the extended, discrete Sb NSs are embedded within the rGO network. Figure 1c,d present transmission electron microscopy (TEM) images characterizing the microstructure of Sb NSs. It is evident that thin and few-layer nanosheets have been achieved through the liquid exfoliation method starting from bulk Sb powder in our experimental work. Figure 1e depicts the morphological structure and energy dispersive spectroscopy (EDS) mapping of the Sb NSs-rGO hybrid, as it can be observed that the Sb NSs are decorated on the rGO layer. Also, EDS data further confirm that the uniformity of Sb NSs-rGO hybrid. Moreover, Figure 1f displays the HRTEM analysis of the Sb NSs-rGO hybrid, where the lattice spacing of 0.32 nm corresponds to the (012) planes of the Sb crystal [6]. It is shown that a well-prepared Sb NSs-rGO hybrid has been constructed. The XRD patterns of the rGO and Sb NSs-rGO hybrid are presented in Figure 1g. The peak positions for samples were well indexed and consistent with crystalline Sb (JCPDS No. 35-0732) [6], implying the formation of crystalline Sb NSs during the hydrothermal processes. While the peak of 25° is ascribed to the rGO [22]. Figure 1h is the Raman spectroscopy, and it is used for confirming the molecular structure and crystalline properties of the material. Raman spectroscopy analysis indicates that both the original Sb nanosheets (NSs) and the Sb NSs-rGO hybrid exhibit characteristic peaks at 110 cm^−1^ and 150 cm^−1^, corresponding to in-plane vibration (Eg mode) and out-of-plane vibration (A_1g_ mode) of Sb nanoparticles, respectively [13,14]. Additionally, a comparative analysis of the pristine GO sample and the hybrid sample reveals that the characteristic G and D peaks for GO and rGO are located at 1585 cm^−1^ and 1325 cm^−1^, respectively. It should be highlighted that the Sb NSs-rGO hybrid exhibit a heightened D peak intensity relative to pristine GO. This is a reasonable result. To sum up, the successful fabrication of the Sb NSs-rGO hybrid can be confirmed.

**Figure 2:**
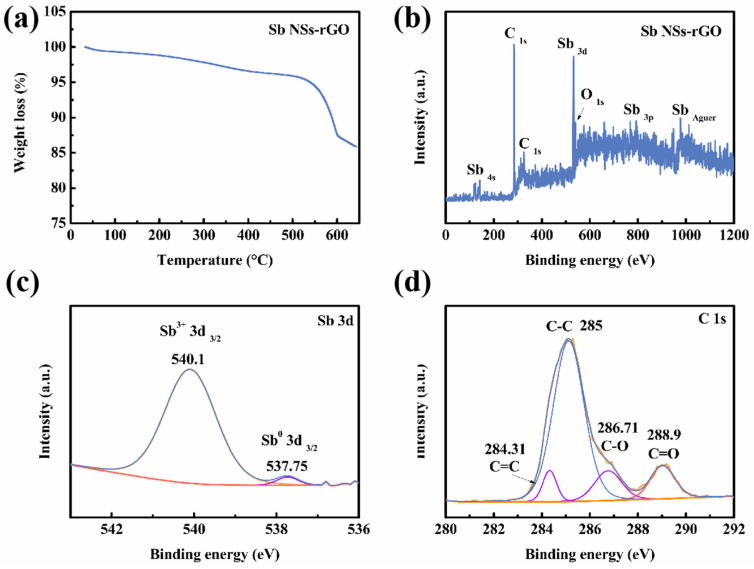
(**a**) TGA curve of the Sb NSs-rGO hybrid under N_2_ atmosphere. (**b**) XPS survey of Sb NSs-rGO hybrid. (**c**) Sb 3d sub-spectra, (**d**) C 1s sub-spectra.

For photoelectrochemical photodetectors, localized heating may occur, especially under illumination and applied bias. Therefore, ensuring the device does not fail due to thermal decomposition under expected operating conditions is highly significant. TGA curves are commonly used as a method for assessing the thermal stability of heterojunction interfaces. Thus, we performed TGA measurement of the Sb NSs-rGO hybrid, as shown in Figure 2a. From the data, it can be observed that weight increase around 300 °C is caused by the decomposition of oxidation of Sb NSs, and the increase around 500 °C is caused by the decomposition of rGO [23]. To obtain more detailed information on the elements on the sample surface, we characterized the sample through XPS analysis for further information. Figure 2b shows the survey spectrum of the Sb NSs-rGO hybrid containing Sb, O, and C elements. It is to be noted that, consistent with the uniformly distributed O elements of TEM-EDS (Figure 1f), there is the presence of a small amount of oxygen on the surface of the nanosheets. Different from the XRD result is that no antimony oxides appear in the Sb NSs-rGO hybrid. A possible explanation could be while minimal surface oxidation is inevitable upon subsequent air exposure, the oxide layer is amorphous and non-crystalline. Figure 2c,d are the Sb 3d and C1s sub-spectra of the Sb NSs-rGO hybrid, respectively. Specifically, Sb 3d peaks in the Sb NSs-rGO hybrid sample, the peak at 540.1 eV and 537.75 eV (Sb 3d 3/2) can be attributed to the valence Sb^3+^ region and Sb metal state (Sb^0^), respectively [6]. While the peak at 284.31, 285, 286.71, and 288.9 eV can be attributed to the C=C, C-C, C-O, and C=O in rGO, respectively [23].

**Figure 3:**
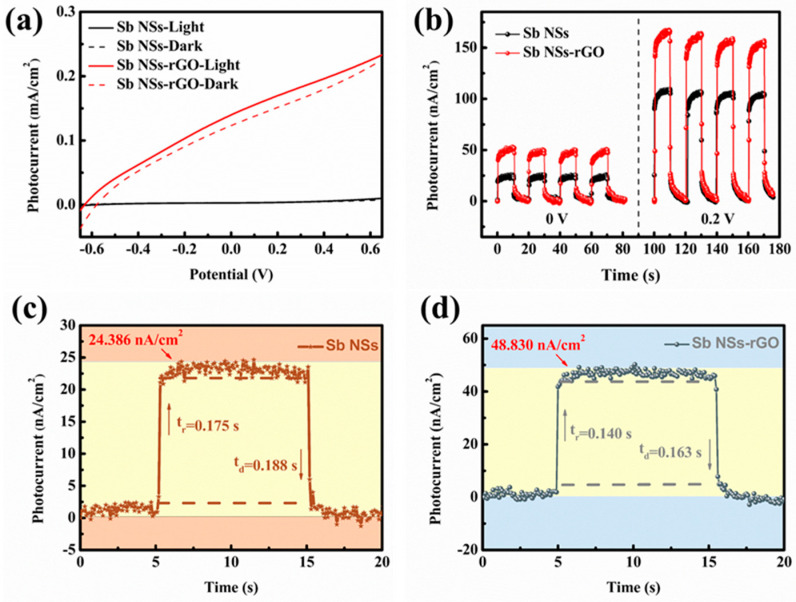
(**a**) LSV measurements of Sb NSs and Sb NSs-rGO hybrid in a 0.5 M Na_2_SO_4_ electrolyte under dark and light irradiation. (**b**) Photocurrent density of Sb NSs and Sb NSs-rGO hybrid in a 0.5 M Na_2_SO_4_ electrolyte under external potential of 0 V and 0.2 V. Normalized photocurrent density versus time associated with the response (τ_res_) and recovery (τ_rec_) time of (**c**) Sb NSs and (**d**) Sb NSs-rGO hybrid photodetectors.

The photoelectrochemical (PEC) performance of the Sb NSs-rGO hybrid was carried out to investigate its photo behaviors. In Figure 3a, the linear sweep voltammetry (LSV) profiles from −0.6 V to 0.6 V for Sb NSs along with the Sb NSs-rGO hybrid are presented. As illustrated in the figure, both the Sb NSs and Sb NSs-rGO hybrids exhibit a positive response behavior upon illumination. The Sb NSs-rGO exhibit a remarkably greater photocurrent enhancement compared to the sole Sb NSs. It is proposed that intermingling rGO enhances the light-responsive properties of Sb NSs. Meanwhile, rGO indicate a poor spectral absorption, as well as Sb NSs increases the intensity of spectral absorption as shown in Figure S1a,b, which indicates the as-prepared Sb NSs-rGO hybrid exhibits an improved absorptive capacity, resulting in numerous electron–hole pairs. Subsequently, the amperometric curve (I-t) test was executed to examine the photo-response performance of both the Sb NSs and the Sb NSs-rGO hybrid. As shown in Figure 3b, the Sb NSs-rGO hybrid demonstrates superior photoresponse properties, with a remarkable enhancement in photoresponse (doubled) compared to Sb NSs under zero bias, which reveals the self-powered photoresponse feature. Moreover, under a 0.2 bias, the photoresponse performance of the Sb NSs-rGO hybrid is approximately 1.8 times that of pure Sb NSs. Notably, rGO exhibit slight photoresponse of 2.34 nA/cm^2^, as shown in Figure S2a, which is due to the restored sp^2^ carbon domains and structural defects facilitate the generation and separation of electron–hole pairs upon light absorption. At the same time, we measured the EIS curves of rGO and the prepared Sb NSs-rGO hybrid under both dark and light conditions, as shown in Figure S2b. It can be seen that rGO, as an excellent medium for charge transport, exhibits a relatively low contact resistance of around 10.4 Ω. However, the Sb NSs-rGO hybrid has a slightly higher contact resistance of around 13.7 Ω. Nevertheless, due to the excellent optical properties of Sb NSs, there are significant resistance fluctuations under both dark and light conditions, indicating that the multi-prepared Sb NSs-rGO hybrid has an improved light response behavior. To investigate their photoresponse behavior under self-powered conditions, I-t tests, as depicted in Figure 3c,d, were carried out to evaluate the photocurrent values and photoresponsivity. The measured photocurrent densities for Sb NSs and the Sb NSs-rGO hybrid photodetectors are 24.386 nA/cm^2^ and 48.830 nA/cm^2^, respectively. Remarkably, the photogenerated current density of the Sb NSs-rGO hybrid photodetector is roughly double that of the standalone Sb NSs photodetector. Furthermore, the photodetector response time is established using a criterion where it is determined at the juncture when 10% of the photocurrent is noted and when 90% of the photocurrent has returned, as described by the given equation [13].

(1) $${I=I}_{0}-Aexp\;\left(-\frac{t}{\tau }\right)$$

(*A*: scaling constant; *τ*: relaxation time constant), respectively. The test results show that the photocurrent response and recover time of the Sb NSs and Sb NSs-rGO hybrid are about 0.175 s/0.188 s and 0.140 s/0.163 s, respectively.

**Figure 4:**
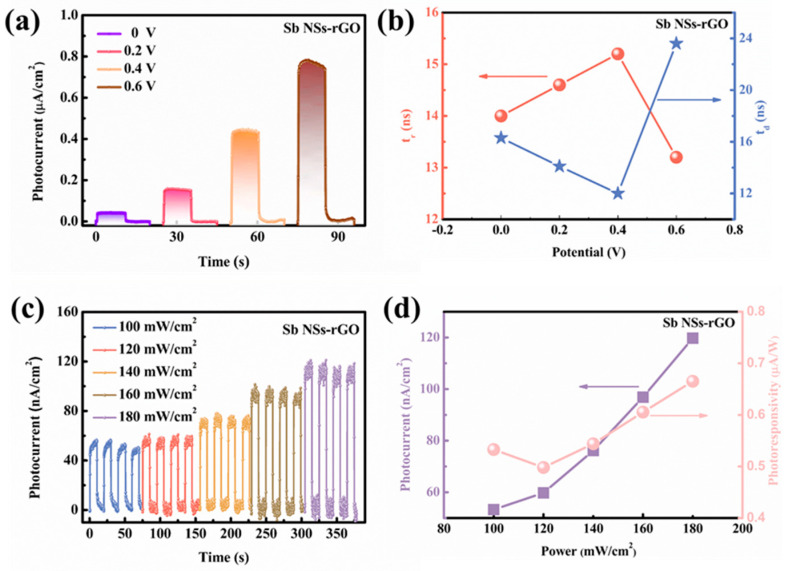
(**a**) Photocurrent density of Sb NSs-rGO hybrid photodetector in the 0.5 M Na_2_SO_4_ electrolyte under external potential from 0 to 0.6 V. (**b**) Dependence of Sb NSs-rGO hybrid photodetector in 0.5 M Na_2_SO_4_ of photocurrent density on response time and relaxation time at different bias voltages from 0 to 0.6 V. (**c**) Photocurrent density of Sb NSs-rGO hybrid photodetector in 0.5 M Na_2_SO_4_ electrolyte under different light intensity (10 to 180 mWcm^−2^). (**d**) The photocurrent fitting curve and calculated responsivity of Sb NSs-rGO hybrid photodetector.

Figure 4a shows the photocurrent density of the Sb NSs-rGO hybrid with increasing applied voltage, where the effect of external bias on the photocurrent is seen. The photocurrent density of the Sb NSs-rGO composite with applied voltage rising from 0 to 0.6 V from 0.05 to 0.76 μA/cm^2^, without changing the switching behavior, is shown. This improvement is because the photogenerated electrons either occur on the photoelectrode surface or are transported to the cathode owing to the action of the electric field, greatly enhancing the photocurrent density of the Sb NSs-rGO hybrid photodetector. In order to clearly reveal the relation of the photocurrent with the response time under different bias voltages, Figure 4b shows the comparison between the photocurrent density and response time under different bias voltage levels. The response time first grows up and then decreases, while the relaxation time first decreases and then increases with the rise in voltage. This behavior is accounted for by the directional dependence of the motion of the charges in the rGO path. Hence, an open-circuit voltage of 0 V was chosen for studying the device through the following PEC tests. Photoresponsivity serves as a critical performance metric for assessing the photo performance capability of photodetectors. As illustrated in Figure 4c, the Sb NSs-rGO hybrid exhibits a strong dependence of photocurrent density on incident light intensity. When the illumination intensity increases from 100 mW/cm^2^ to 180 mW/cm^2^, the photocurrent density rises significantly from 48.832 nA/cm^2^ to 118.265 nA/cm^2^. Besides, to evaluate the sensing performance of photocurrent as a function of light intensity (Figure 4d), the concept of responsiveness (R) is presented, which characterizes photocurrent produced in response to incident light across the effective area. The formula for *R* is defined as follows:

(2) $$R=\frac{\left(I_{light}-I_{dark\;}\right)}{PS}$$

where *I_light_ − I_dark_* is photocurrent, *P* is the light illumination intensity, and *S* refers to the effective illuminated area. The calculated photoresponsivity of the Sb NSs-rGO hybrid is 0.68 μA/W. A comparison of the PEC photodetector parameters for 2D materials have summarized in Table 1, indicating that the Sb NSs-rGO hybrid shows a great photo-response of other 2D-based materials hybrids.

**Figure 5:**
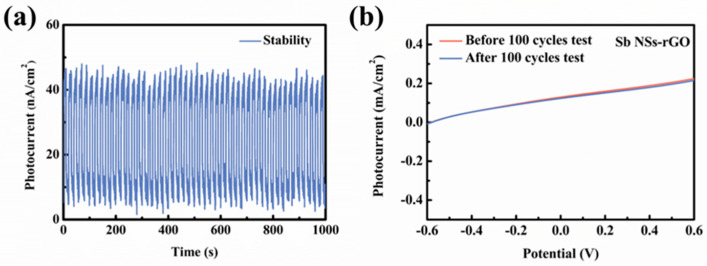
(**a**) Cycle stability test of Sb NSs-rGO hybrid photodetector in 0.5 M Na_2_SO_4_ electrolyte at a basis potential of 0 V. (**b**) LSV test characteristic curve of the NSs-rGO hybrid photodetector before and after 100 cycles.

The stability of Sb-NSs-rGO hybrid photodetector in the process of photo switching behavior directly affects the practical application value. Therefore, it is necessary to conduct tests and evaluate the stability of PEC-type photodetectors on the basis of Sb NSs-rGO hybrids. It can be seen from Figure 5a that after light “on and off” tests for 1000 s, with minor fluctuations around 47.5 nA/cm^2^, there was great stability. No detectable change was observed in its I–V curve until 1000 s was scanned, which indicates that the Sb NSs-rGO hybrid has very good stability in the 0.5 M Na_2_SO_4_ solution. The curve of linear scanning voltammetry (LSV) characteristics of photodetector did not change noticeably in the course of 100 scanning cycles before and after scanning, as displayed in Figure 5b. This shows very good stability of the Sb-NSs-rGO hybrid PEC photodetector. The result implies that the Sb-NSs-rGO hybrid photodetectors exhibits excellent stability.

**Figure 6:**
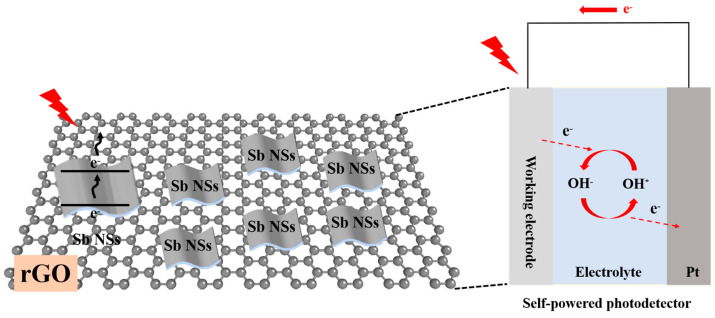
Self-powered mechanism of the prepared Sb NSs-rGO hybrid PEC-type photodetector.

The self-powered mechanism of the prepared Sb NSs-rGO hybrid PEC-type photodetector as shown in Figure 6. When incident light irradiates, the photogenerated electrons transition from the valence band of Sb NSs to the conduction band, while rGO provides a high-conductivity pathway for electron transport, while the heterojunction interface promotes the separation of photogenerated carriers. The built-in electric field-driven by the generated bias potential results from the semiconductor potential barrier difference between sbNSs-rGO and the electrolyte. This spatial separation of charge carriers effectively suppresses the recombination of electron–hole pairs. Similar to the operation mechanism in a Schottky-type photodetector, this electrochemical reaction process spontaneously generates free electrons and establishes a stable photocurrent loop. The efficient charge separation and rapid carrier transport within the heterojunction contribute to the enhanced photoresponse performance, including improved responsivity and faster response dynamics.

## 4. Conclusions

In summary, a self-powered PEC photodetector derived from the Sb-NSs-rGO hybrid was successfully synthesized by simple liquid exfoliation followed by a hydrothermal process. The morphology and microstructure characterization showed that the liquid-exfoliated Sb-NSs possess good crystallinity and exhibit a strong anchoring effect on the rGO. The combined coupling together with rapid photogenerated electron–hole pair separation can not only promote the interface fast efficient charge transfer, but can also promote the performance of the photoresponse of the Sb-NSs-rGO hybrid photodetector. Our work shows that the prepared Sb-NSs-rGO hybrid can provide an interesting candidate for the self-powered PEC-type photodetector.

## Figures and Tables

**Table 1:** Comparison of PEC photodetector parameters for 2D materials.

Photodetectors /Materials	Method	Response/Recovery Time (ms)	Responsivity	Photocurrent Density (µA/cm^2^)	Test Parameters	Ref.
MoS_2_–Se Nanocomposites	Hydrothermal synthesis	120/420	38.3 μA/W	/	100 mW/cm^2^	[24]
SnSe/SnSe_2_	Hydrothermal synthesis	200/600	442 μA/W	4.42	520 nm, 10 mW/cm^2^	[25]
WS_2_ nanotubes	Chemical Vapor Deposition	20/20	17.39 A/W	0.009	532 nm, 1.42 mW/cm^2^	[26]
2D WS2-graphene	Liquid-exfoliation and hydrothermal synthesis	1200/900	/	1.4	30 mW cm^−2^	[27]
Sb NSs-rGO	Liquid-exfoliation and hydrothermal synthesis	140/163	0.68 μA/W	1.96	100 mW/cm^2^	This work

## Data Availability

The corresponding author, I.K.R., can provide the data, codes, and materials required for the completion of this manuscript.

## Supplementary Materials

The following supporting information can be downloaded at: https://www.mdpi.com/article/10.3390/nano15171355/s1, Figure S1. (a) UV-vis spectra of rGO and (b) as-prepared Sb NSs-rGO hybrid. Figure S2. (a) Normalized photocurrent density of rGO. (b) EIS curves of rGO and the as-prepared Sb NSs-rGO hybrid under dark and light.
