# Supplementary material for: Liquid-Exfoliated Antimony Nanosheets Hybridized with Reduced Graphene Oxide for Photoelectrochemical Photodetectors
Source: Nanomaterials (Basel). 2025 Sep 3;15(17):1355. doi: 10.3390/nano15171355 (PMC12430401; doi:10.3390/nano15171355)
Supplement: Supplementary file 1 [file nanomaterials-15-01355-s001.zip › nanomaterials-3756845-supplementary.pdf]

Supporting information

Liquid-Exfoliated Antimony Nanosheets Hybridized with Reduced Graphene Oxide for Photoelectrochemical Photodetectors

Gengcheng Liao [1,†], Sichao Yu [1,†], Jiebo Zeng [2], Zongyu Huang [2,3], Xiang Qi [2,*], Jianxin Zhong [1] and Long Ren [1,*]

[1] Institute for Quantum Science and Technology, Shanghai University, Shanghai 200444, China
[2] Hunan Key Laboratory of Micro-Nano Energy Materials and Devices, Laboratory for Quantum Engineering and Micro-Nano Energy Technology, School of Physics and Optoelectronic, Xiangtan University, Xiangtan 411105, China
[3] Hunan Key Laboratory of Two Dimensional Materials, Hunan University, Changsha 410082, China
* Correspondence: xqi@xtu.edu.cn (X.Q.); renlong@whut.edu.cn (L.R.)
† These authors contributed equally to this work.

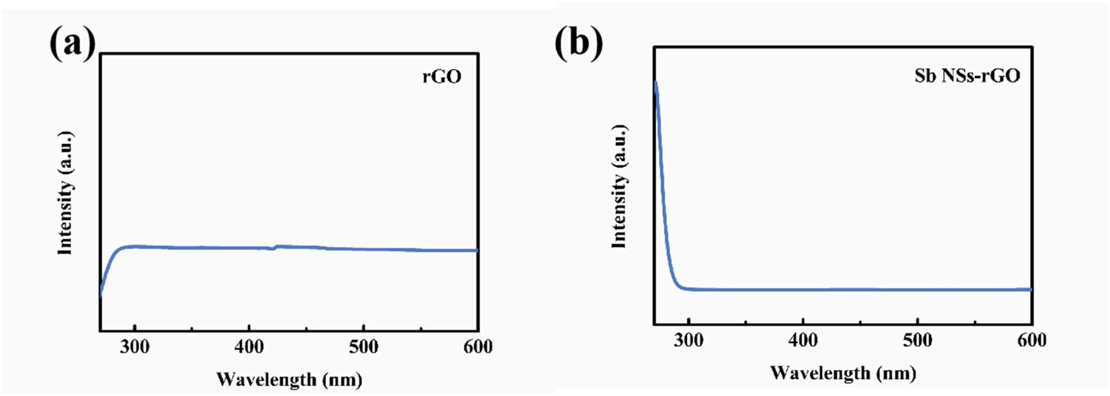

Figure S1. (**a**) UV-vis spectra of rGO and (**b**) as-prepared Sb NSs-rGO hybrid.

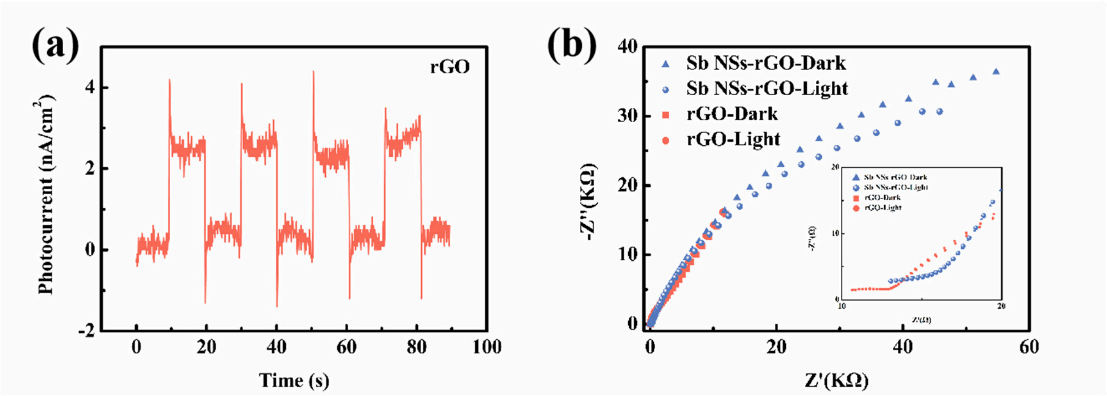

Figure S2. (**a**) Normalized photocurrent density of rGO. (**b**) EIS curves of rGO and the as-prepared Sb NSs-rGO hybrid under dark and light.
